# Different Methods for Long-term Systematic Assessment of Challenging Behaviors in People with Severe Intellectual Disability

**DOI:** 10.3389/fpsyg.2017.00017

**Published:** 2017-01-19

**Authors:** Candida Delgado, Rodrigo G. Gonzalez-Gordon, Estívaliz Aragón, Jose I. Navarro

**Affiliations:** ^1^Department of Psychology, University of CadizCadiz, Spain; ^2^Special Education Center, AFANAS-JerezCadiz, Spain

**Keywords:** challenging behaviors, functional assessment, observer XT, intellectual disabilities, observation procedures

## Abstract

The aim of this study was to compare the advantages and disadvantages of different behavioral assessment procedures with the purpose of design a long-term assessment procedure that brings together the benefits observed. The study involved four adults with severe and profound intellectual disabilities and severe behavioral problems. A behavioral assessment has been carried out with Scatter Plot, Antecedent-Behavior-Consequence record sheets and, finally, The Observer XT. We conducted a comprehensive assessment of challenging behavior of each participant, by reviewing bio-psychosocial records, interviewing family and direct caregiver staff, as well as direct observation. A large inter-subject behavior variability and instability of intra-subject behavior were found, raising questions about the election of the period to evaluate. The first evaluation level highlighted information about stability of behavior, the second level stand out the context in which it occurs and the third level detailed the different topographies of each behavioral category. The results suggest the desirability of a continuous assessment by combining these three levels for adjusting to the specific characteristics of behavior. We suggest the need of designing a single behavioral assessment procedure that includes the benefits observed in each of the instruments used.

## Introduction

Challenging behaviors (self-injury, physical, or verbal aggression, destruction of objects, stereotyped behaviors, and social-offensive behaviors) are especially prevalent in adults with severe intellectual disabilities (ID), becoming one of the biggest challenges for researchers (Lowe et al., 2007) and for service providers. It has been estimated that the prevalence of challenging behaviors (CB) in people with learning disabilities has ranged from 10% (Emerson and Einfeld, 2011) to 15% (Holden and Gitlesen, 2006), increasing the likelihood of CB with higher levels of ID (Janssen et al., 2002).

In addition, CBs are more likely to be presented in people who are non-verbal or who have particular difficulty with receptive or expressive communication (Holden and Gitlesen, 2006). Depending on the severity, CB in people with ID can be a serious threat to their health and safety, restricting the opportunities to integrate into community, producing stress in their families and caregiver staff and drastically reducing opportunities to carry out an educational intervention. They also are in higher risk of abuses from those who support them (Verdugo and Gutiérrez, 2009; Poppes et al., 2010). Specifically, the presence of stereotyped behavior in the persons can interfere with their adaptive responding and learning opportunities, cause health concerns and injuries, hamper their social image and their overall acceptance by others (Holburn et al., 2004).

Challenging behaviors refers to behaviors which involve significant risks to people's physical well-being or act to markedly reduce access to community settings (Emerson and Einfeld, 2011). This justifies the importance of early and effective interventions to change behavior. Horner et al. (2002) demonstrated that early behavioral intervention could reduce 80–90% of CB. However, the lack of intervention against CB tends to adversely affect people with disabilities, causing behavioral problems persistence in the individual repertoire and limiting social, environmental, and educational experiences in adulthood (Murphy et al., 2005).

To implement an effective intervention, we must first conduct a functional behavior analysis to gather and synthesize information, to define the behavior problem, to identify the behavior consequences and describe the environmental context associated with high and low rates (Repp and Horner, 1999). This methodology has become the main strategy for behavioral assessment of current approaches (Hanley et al., 2003; Brosnan and Healy, 2011). In any case, the behavior problem analysis always requires a comprehensive assessment of their frequency, duration, intensity, and context.

Functional analysis has become one of the widely used resources between behavioral specialists to assess CB (Iwata et al., 1982, 1994; Phillips and Mudford, 2011; Shabani et al., 2013). However, most educational and clinical settings do not implement experimental analysis to assess the behavior before starting a treatment because of the practical difficulties. Realistic difficulties reported by some practitioners are: low probability for observing the behavioral problem with limited observation timing, the requirement for caregiver staff specific training to carry out the analysis, and the human resources required by an appropriated functional analysis of behavior (Matson, 2012).

As a result, a functional descriptive assessment with direct observation in natural contexts to assess behavioral problems in clinical and educational setting has been established. One of the descriptive functional evaluation methods is the Antecedent-Behavior-Consequence (ABC). This is a frequently used record form in non-experimental observation context for functional behavioral assessments. Each occurrence of a specific event (and the antecedents and consequences) is marked. Data reveal the events that are closely related to the target behavior (Sasso et al., 1992). O'Neill et al. (1997) designed an observation sheet for the functional evaluation containing eight sections: participant's information, date, observation time intervals, specific behavior problems, predictors, functions, and current consequences perceived.

Scatter Plot analysis is other descriptive functional assessment method to help in identifying patterns of responses in natural settings (Borrero and Vollmer, 2002). The main advantage of Scatter Plot analysis is the identification of periods of time during which the behavior occurs. This information can be useful for planning the functional evaluation periods registered by ABC in order to get evidences about the functional role of any non-adaptive behavior (Matson, 2012).

Although traditionally live observation methods with paper-and-pencil registration sheets have been used, new technologies observation using electronic devices and specific software also have been used. The Observer XT is a tool for researchers and practitioners, recently used in observation with autism and intellectual disability children (Meirsschaut et al., 2011; Mossman, 2011; Hutman et al., 2012).

However, few methodological studies evaluating advantages and disadvantages of those three functional assessment methods have been disseminated. Phillips et al. (2014) conducted a study with 15 observers recording response rates from 10 video clips using one out of three input formats: keyboard (laptop), touch screen (digital assistants personal), or paper-and-pencil. The results showed that although the electronic record had the potential to be more accurate and more reliable than paper-and-pencil method, this was far from being conclusive. The analysis of errors registered determined the necessity to improve data accuracy and reliability when either method is used. Other study comparing both electronic and traditional paper-and-pencil methods, recording responses from four children with autism (Tarbox et al., 2010). The results showed that the electronic data collection was more time consuming than paper-and-pencil.

Therefore, when resources do not permit use an appropriated experimental analysis of behavior assessment (e.g., non-appropriated caregivers training), or behavioral problem rates are too low, and requires an excessive amount of observations periods in a non-natural settings, or the behavior is disturbed by an intrusive medical treatments (Cox and Virues-Ortega, 2016), descriptive and indirect functional assessment methods can provide helpful information (Matson, 2012).

The aim of this paper is to show three different methods of objective assessment and subsequent analysis of the behavior of four adults with severe ID and CB added, using a triangular system, which analyzes the person, his environment and interactions. All this through an evaluation at three levels: (1) general assessment through Scatter Plot register, (2) evaluation by ABC record sheets, and (3) direct observation of behavior through video recording and analysis using The Observer XT. Depending on the assessment preferred, different variables may be implemented to improve the reliability and validity of the analysis: Establishing a baseline period of observation, disclosure, or withholding the behavioral function, and whether or not a cost-benefit correction is allowed.

## Materials and methods

### Participants

This study involved four males with severe and profound ID and serious CB added, aged between 32 and 42 years. Users with the most serious CB were chosen. The sample was selected from a special education center. This institution belongs to a non-governmental and non-profit organization, with 45 years' experience in helping people with severe ID. CBs were operationally defined and the different topographies of each behavioral category were identified in each participant. Participant's names are pseudonyms.

Case 1. Ivo, showed: (a) self-injury behavior (banging his head, belly, chest, leg, hand, and arm), (b) physical aggressive behavior (kicking, head banging, hitting, or punching), (c) stereotyped or repetitive behaviors (wandering, rocking and moving an object), (d) social-offensive behaviors (urinating in the classroom, get undressed in public), (e) damage or destruction of objects (throw materials) and (f) disruptive behavior (escape from classroom). All these behaviors appeared very frequently, being present, and having psychotropic drugs prescribed chronically since childhood. Neurosurgical intervention (bilateral criohipothalamotomy) was practiced twice to reduce CB. After neurosurgical and behavioral interventions (such as positive and negative reinforcements, and time out), behaviors problem decreased, but never disappeared.

Case 2. Jorge, showed: (a) self-injury behavior (beating his chest, face, leg, and head), (b) physical aggressive behavior (slaps, punches and kicks), (c) damage or destruction of objects (throw material, hits the table, window and mat) (d) stereotyped or repetitive behaviors (waving a handkerchief, yelling), and (e) compulsive acts (turn on/off radio or TV, opening/closing windows or doors, order materials, turning lights on/off, separate legs crossed, pull up his socks, and to close containers). Compulsive acts occurred more frequently than other behaviors, which were exhibited with higher intensity. CBs have been present and have received chronic psychopharmacological treatment since childhood.

Case 3. Vicente showed: (a) self-injury behavior (head banging, clawing face), (b) physical aggressive behavior (slaps, pinches, scratches, pulls hair, clothing, and ear) and (c) stereotyped or repetitive behaviors (rocking, wandering, waving an object and rubbing the palate with the thumb). These behaviors have resulted in wounds or scratches on his face or to others around him. They occurred very frequently, have been present and have received chronic psychopharmacological treatment since childhood.

Case 4. David showed: (a) verbal aggressive behavior (insulting others), (b) physical aggression behaviors (jump over others around him, pinching or gripping tightly to others), (c) self-injury behaviors (drops to the floor with violence, banging his head against the wall). These behaviors have been present and have received chronic psychopharmacological treatment since childhood. There have been several attempts to treat the CB by physical restraints, medication change and modification of the environment with engaging activities. However, although there was a reduction in aggressive behavior, he still has a high frequency.

In the study, parents were individually informed of the entire procedure which would take place, stating verbal consent on the adequacy of planned activities. Written informed consent was obtained from the parents of all participants. No ethical approval was required for this type of research, in accordance with the legislation in Spain.

### Instruments

1. *Scatter Plot*. The CB record sheet was extracted from the computer system for tracking bio-psychosocial people with intellectual disabilities (García González-Gordon et al., 2005). This is a computerized form to record the occurrence of different adaptive and CB. We focused only on the CB record sheet, which contains monthly data on the days when participants were involved in any CB episode. Behaviors described fit the classification provided by the Inventory for Client and Agency Planning (ICAP, Montero, 1996). The CB recorded with the Scatter Plot were self-injury, physical, and verbal aggression, destruction of objects, and socially offensive behaviors. With these records, we obtained continuous information about the evolution of CBs in which the user was involved, allowing us to verify the more or less frequency periods.
2. *ABC record sheet*. ABC is a paper-and-pencil record sheet where problem behaviors occurrence, antecedents and consequences were registered. This was used to collect the episodes of CBs, and it is based on the model proposed by O'Neill et al. (1997). Registers were individually made, taking into account the characteristics of each participant, based on information obtained through interviews and clinical records. This register system allowed us to determine the frequency of CBs, patterns of occurrence and the absence of such behavior in the registration periods of time.
3. *The Observer XT version 10.0. Noldus Technology* (Grieco et al., 2011). This software allowed observe, record and analyze human behavior through digital video tapes. The introduction of data requires a previous design of a template that includes topography details for each behavioral category and the contextual conditions that are considered relevant to include in the project. For example we defined the stereotype behavior as a state event (how long participants were involved in this CB) and self-injury behavior as a point event (frequency of the CB). The recording of data in the observation module displays the video while coded manually behaviors, providing the possibility of slowing down the video, making comments, display filtered behaviors, and exclude parts of the video which analysis was not useful. With these video files we can check behaviors in a systematic and exhaustive way, rigorously examine the frequency, duration and intensity of each selected behavior, facilitating data entry, offering the possibility of issuing reports and statistical charts quickly. This tool was selected because it offers the possibility to comprehensively analyze as often as necessary video files, and be used for live observation (Kahng et al., 1998).

The Observer XT is rarely used in clinical settings, but frequently utilized for researching proposes. However, ABC sheets and Scatter Plot are procedures frequently used in clinical and researching settings. All records were performed by qualified personnel trained in behavioral observation of severely affected people.

### Procedure

Using a descriptive design, the study recorded several CBs (dependent variable) from participants with ID with different assessment tools (independent variable). In the first phase of the study, we gathered information on participants, through bio-psychosocial records, re-viewing the results of CB assessment with ICAP (Montero, 1996) and DASH II (Salvador et al., 2002) in order to know their repertoire and intensity in the past. We also administered a semi-structured interview to direct-caregiver staff and family, collecting information concerning the history of CB, their development and interventions performed so far. Finally, we carried out a live observation of behavior problems of each participant at three different levels:

1. We asked a well-trained direct-caregiver staff to daily record in the Scatter Plot all previously defined CBs episodes. We could obtain a continuous verification of behavior's occurrence. The register also included absence's days at Out-Patients Unit as well as events that could lead to major changes in participants' routine. During this process, the well-trained staff registered on a manual sheet if an episode of self-injury behavior, physical aggression, social-offensive behavior or destruction of objects had occurred during the day. Subsequently, this information was registered to the computer Scatter Plot. We analyzed Ivo and Jorge “behaviors for 33 months, and David” behaviors for 18 months. Data from Vicente's behaviors were not analyzed for lack of reliability. Information was organized by years calculating the percentage of CB occurrence per each participant.
2. On a second level we used the ABC record sheets, containing individualized and more specific information about those CBs in which the user was involved and the possible antecedents and consequences that could explain their maintenance. The ABC record sheets were conducted with three participants (Ivo, Jorge, and Vicente) over 15 days of recording. Each participant was observed by a specialist trained in behavioral analysis. Before configuration of ABC record sheets, operational definitions were obtained from information provided by family members and direct-caregiver staff. Then prevalence of different topographies of each behavioral category was calculated. In addition, we computed the frequency of the different antecedents and consequences, obtaining quantitative information about the hypothesized functions that might explain CB.
3. At the third level, an external expert in applied behavior analysis, continuously observed (ABC procedure) and videotaped CB of four participants in a natural environment during 15 days. They participated on different academic or daily tasks, at the Out-Patients Unit. Then all sessions were analyzed by The Observer XT. We recorded the frequency of the different topographies of each behavioral category, including information concerning the context in which they operate, the days of the week and people surrounding the participant. Data collection was carried out for 15 alternative daily 120 m sessions each. The observation sessions with the Observer XT were conducted by two observers specifically trained in the software practice, carrying out an IOA 33% of the observation sessions (10 h) in two of the participants (Ivo and Jorge).

## Results

The assessment of CB was held through the three levels (Scatter Plot, ABC, and The Observer XT). The results include a descriptive analysis of the three procedures used in the study. We analyzed with the Scatter Plot reports the days in which participants were involved or not in a CB's episode through years 2009, 2010, and 2011. As seen in Table 1, there was an extensive variability between behaviors, but not all participants presented verbal aggression or social-offensive behaviors. A large variability was found as well-considering the frequency of shared behavioral categories. Similarly, the dispersion of CB's episodes over time makes assessment more difficult, as seen in the case of David. CB's episodes were not very frequent over the 431 days recorded.

**Table 1:** **Days in which participants involved in challenging behaviors (CB) recorded with the Scatter Plot**.

**Challenging behaviors**	**Ivo**		**Jorge**		**David**	
	**CB**	**No CB**	**CB**	**No CB**	**CB**	**No CB**
Physical aggressive behavior	256 (36%)	446 (64%)	135 (33%)	279 (67%)	86 (20%)	345 (80%)
Verbal aggressive behavior	–	–	–	–	77 (18%)	354 (82%)
Self-injury	577 (82%)	125 (18%)	34 (8%)	380 (92%)	27 (6%)	404 (94%)
Destruction of objects	109 (16%)	593 (84%)	335 (81%)	79 (19%)	1 (0%)	430 (100%)
Social-offensive behaviors	63 (9%)	639 (91%)	15 (4%)	399 (96%)	–	–

**Figure 1:**
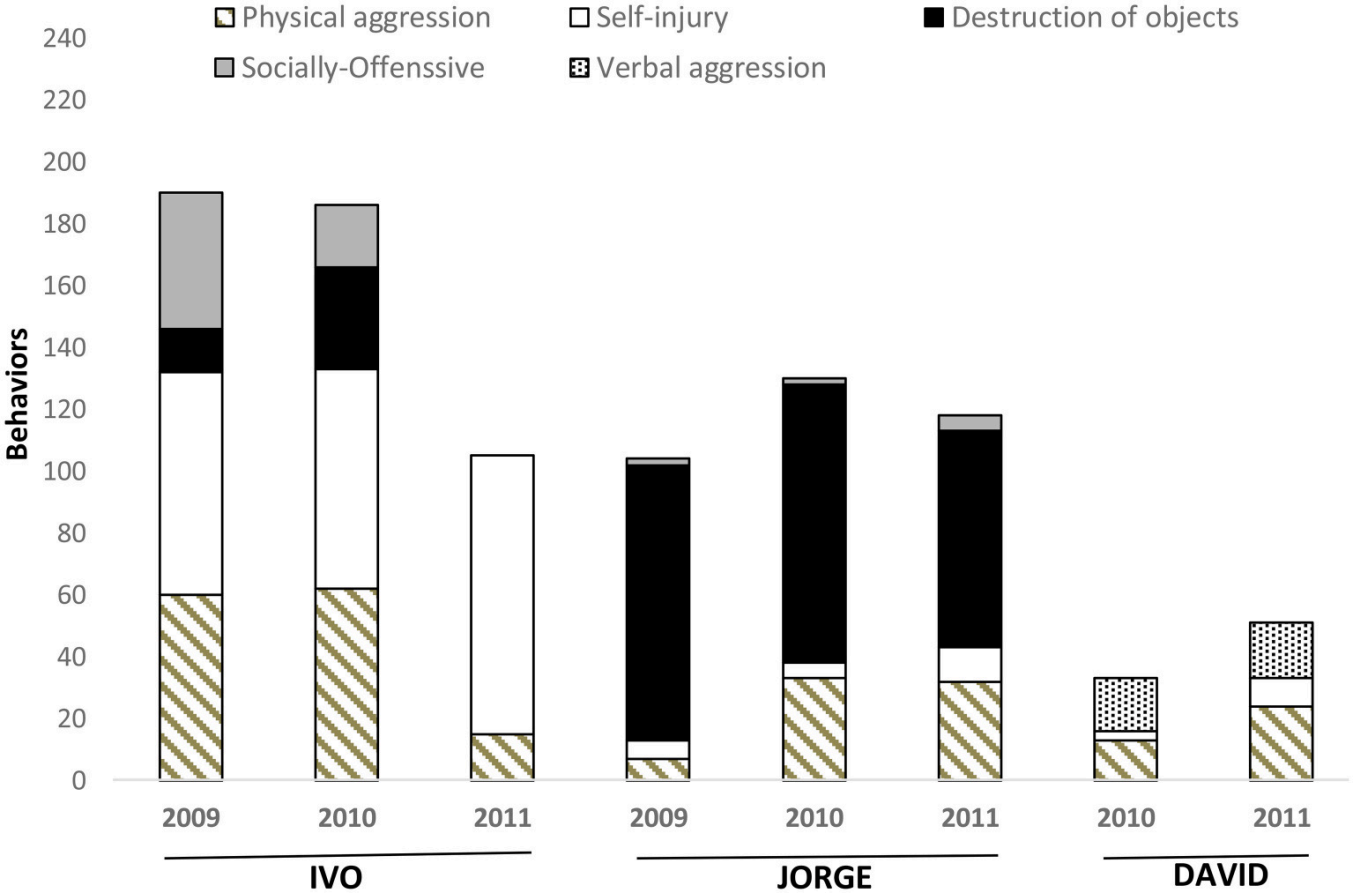
**Percentage of days in which Jorge, David, and Ivo were involved in challenging behavior episodes, evaluated by Scatter Plot through**.

The results of Scatter Plot's assessment of Ivo, Jorge, and David for three consecutive years in the first two cases, and two consecutive years in David are presented in Figure 1. This method allowed track progression of behavior through long periods. Ivo sequence shows a decrease of social-offensive behaviors and destruction of objects, until its complete disappearance. A reduction of 47% in the presence of physical aggressive behavior and increasing 19% for self-injury was also noted. Jorge achieved more stability in behavior over time. The behavioral category most frequently observed through 3 years was destruction of objects (83%), physical aggressive behavior (24%) and self-injury (7%). David's assessment using the Scatter Plot suggested that his behavior was highly unstable, making more difficult direct observation because of the unpredictability in the occurrence of episodes. As Figure 1 show, an increasing 11% for physical aggressive behavior and 6% in the present of self-injury behavior was noted.

Table 2 shows the comparison of CB's frequency by Jorge and Vicente using two different procedures, ABC record sheets and The Observer XT simultaneously recorded. With both procedures 30 h were evaluated during 15 days along 2 consecutive months. Evaluation with both procedures was conducted in two periods of 15 days. For example, in the case of Vicente, with the ABC procedure there was only one self-injury episode, having recorded 101 episodes with The Observer XT. Alternatively, in the case of Jorge, with the ABC procedure 16 hitting the table episodes were observed, while with The Observer XT no episodes were registered. These results show how different frequencies can be observed depending on the period evaluated and method used (see Table 2). An interobserver agreement was calculated for 33% of the Observer XT Jorge and Ivo' records. The IAO average range was between 91 and 99% for each participant (Cohen's *K* = 092; *p* < 0.001).

**Table 2:** **Jorge and Vicente's challenging behaviors frequency observed with ABC record sheets and The Observer XT**.

		**ABC recording sheets**	**The Observer XT**
**JORGE CHALLENGING BEHAVIORS**			
Compulsive acts		159	142
	Turn on/off light	7	47
	Open/close the door	42	43
	Open/close the window	32	11
	Order materials	47	23
	Turn on the radio	13	1
	Separate crossed legs	18	9
Self-injury		25	15
	Hitting his face	21	7
	Banging his head	4	0
	Hitting his leg	0	7
	Hitting his chest	0	1
Physical aggressive behaviors		14	19
	Hitting others	9	16
	Kicking others	5	3
Damage or destruction of objects		18	15
	Hitting the table	16	0
	Hitting the window	1	11
	Hitting the mat	0	2
	Hitting the wall	1	0
	Throwing material	0	2
**VICENTE CHALLENGING BEHAVIORS**			
Self-injury		1	101
	Banging his head	1	100
	Clawing his face	0	1
Physical aggressive behaviors		60	55
	Pulling clothes	21	19
	Hitting others	4	16
	Pinching others	24	12
	Pulling others hair	6	5
	Scratching others	3	2
	Pulling others ear	2	1

The assessment carried out using The Observer XT allowed us to analyze different topographies of each behavioral category, providing us information about frequency, mean, standard deviation, and the rate per minute for each CB (see Table 3). It also allowed compare the differences between participants, considering CBs categories and specific topographies. All participants shared some CB categories (self-injury, stereotyped behaviors, and physical aggression). However, there were differences between participants for disruptive behavior and compulsive acts. For example, Jorge was the only case with compulsive acts. There were also large differences in frequency of CB episodes between subjects.

**Table 3:** **Descriptive data of the challenging behaviors presented by Ivo, Jorge, and Vicente obtained by The Observer XT**.

	**Results obtained by The Observer XT**					
	***N***	**Min**.	**Max**.	**Mean**	**SD**	**Rate/min**
**IVO CHALLENGING BEHAVIORS**						
Total self-injury behaviors	391					
Hitting his leg	205	0	53	13.67	18.02	0.17
Banging his head	102	0	22	6.8	9.03	0.09
Hitting his hand	45	0	19	3	6	0.05
Hitting his arm	20	0	15	1.33	3.89	0.02
Hitting his belly	9	0	4	0.6	1.18	0.008
Hitting his face	6	0	3	0.4	1.06	0.005
Hitting his chest	4	0	3	0.27	0.8	0.003
Total physical aggression behaviors	83					
Hitting others	61	0	20	4.07	5.81	0.05
Kicking others	13	0	4	0.87	1.51	0.01
Head banging others	5	0	3	0.33	0.9	0.004
Punching others	4	0	1	0.07	0.26	0.002
Total disruptive behaviors	35					
Escaping from work room	35	0	9	2.33	2.72	0.03
Damage or destruction of objects	27					
Throwing material	27	0	6	1.8	2.43	0.02
Total socially-offensive behaviors	2					
Being naked in public	1	0	1	0.07	0.26	0
Urinating in classroom	1	0	1	0.07	0.26	0
Total stereotyped behaviors	256					
Wandering	134	0	47	7.87	12.17	0.089
Rocking	110	0	33	7.33	9.5	0.073
Waving an object	12	0	12	0.8	2.83	0.008
**JORGE CHALLENGING BEHAVIORS**						
Total stereotyped behaviors	303					
Shake scarf	246	2	49	16.4	11.48	0.18
Shout	57	0	13	3.8	4.07	0.08
Total compulsive acts	144					
Turn on the light	33	0	9	2.2	2.15	0.03
Close the door	38	0	6	2.53	1.81	0.04
Order materials	20	0	4	1.33	1.29	0.23
Turn off the light	14	0	3	0.93	1.34	0.02
Separate legs crossed	9	0	3	0.6	1.06	0.03
Pulls up socks	9	0	2	0.6	0.83	0.02
Close the window	8	0	4	0.53	1.13	0.34
Open the door	5	0	4	0.33	1.05	0.03
Open the window	3	0	3	0.2	0.78	0.03
Open curtains	2	0	2	0.13	0.52	0.02
Turn on the radio	2	0	2	0.13	0.52	0.02
Close the pot	1	0	1	0.07	0.26	0.01
Total physical aggression behaviors	19					
Hitting others	16	0	5	1.14	1.56	0.03
Kicking others	3	0	2	0.21	0.58	0.02
Total damage or destruction objects	14					
Hitting the mat	10	0	5	0.73	1.43	0.04
Hitting the window	2	0	2	0.13	0.52	0.02
Throwing material	2	0	1	0.13	0.35	0.04
Total self-injury	12					
Hitting his face	7	0	3	0.5	0.86	0.01
Hitting his leg	4	0	3	0.5	1.09	0.03
Hitting his chest	1	0	1	0.7	0.27	0.001
**VICENTE CHALLENGING BEHAVIORS**						
Total stereotyped behaviors	998					
Rubbing his palate with his thumb	421	2	84	23.39	20.21	0.36
Rocking	240	2	28	12.71	8	2321.34
Waving an object	263	6	52	15.41	12.67	0.22
Wandering	74	0	13	4.11	4.21	677.62
Total self-injury	101					
Banging his head	100	0	46	5.56	11.16	0.16
Clawing his face	1	0	1	0.06	0.24	0.04
Physical aggression	54					
Pulls clothes	19	0	6	1.06	1.59	0.04
Hitting others	16	0	5	0.89	1.49	0.04
Pinching others	12	0	6	0.67	1.41	0.03
Pulling others hair	5	0	3	0.28	0.75	0.29
Clawing others	2	0	2	0.11	0.47	0.44

*30 hours of observation*.

This software also facilitated recording of the antecedents and consequences that may functionally explain the behavior. It allowed us to obtain results about the frequency during behavioral assessment (see Table 4). In the case of Ivo, most of the CBs episodes should functionally explained because of lack of received attention, or because he was interrupted when he was doing something reinforcing. We can also consider that self-stimulation had a significant functional role in most self-injury episodes.

**Table 4:** **Relationship between the antecedents, challenging behaviors and the consequences observed in episodes presented by Ivo**.

	**Self-injury**							**Physical aggression**			**Disruptive**		**Total**
	**Hitting his leg**	**Banging his head**	**Hitting his hand**	**Hitting his arm**	**Hitting his face**	**Hitting his belly**	**Hitting his chest**	**Hitting**	**Punching**	**Kicking**	**Escaping**	**Throwing material**	
**ANTECEDENTS**													
Not receiving attention	9	6	13	3	2	2	2	2	2	1	7	1	50
Interruption	1	1	5	–	–	–	–	7	1	2	1	1	19
Transition	–	–	–	–	–	–	–	–	–	–	1	–	1
Difficult task	1	2	–	–	–	1	–	2	–	–	–	–	6
Receiving an instruction	1	1	1	–	–	2	–	–	–	–	1	1	7
Communication attempt	–	–	–	–	–	–	–	1	–	–	–	–	1
**CONSEQUENCES**													
Get self-stimulation	7	6	12	3	2	2	–	1	–	–	–	–	33
Get object/activity	1	1	3	–	–	–	–	2	–	–	2	–	9
Gain attention	1	–	1	–	–	1	1	2	2	1	8	1	18
Avoid instruction	1	–	2	–	–	2	–	2	–	–	–	1	8
Avoid activity	1	2	–	–	–	1	–	1	1	–	–	1	6
Avoid people	1	1	1	–	–	–	–	3	1	2	–	–	9

## Discussion

The aim of this study was to compare advantages and disadvantages of different behavioral assessment procedures. The main practice objective was to design a system for long-term assessment that brings together the benefits observed. It has been carried out a behavioral assessment by a first general level (Scatter Plot), a more specific level (ABC) and, finally, a very detailed level (The Observer XT).

The results showed that CB's episodes were unstable. They varied over time, subsisting periods of high rates of behavior problems and periods in which only a few number of episodes were registered. Based on these results, selection of most appropriate recording system depend on the stability of episodes of CB. When episodes are infrequent but high intensity, occurring sporadically, the Scatterplot seems more appropriate registering procedure. For such profiles, The Observer XT is not useful for recording the behavior when no events occur because it does not allow us to describe the environmental context associated with high rates of behavioral problems. Disruptive behaviors had a great variability. There were periods of observation where the CB did not show up. In these cases, the recommended registration procedure seems Scatterplot. However, for behaviors with very high frequency the Observer XT could be a better choice, since they can be accurately recorded every time the CB appears, and also the consequences of such behavior.

The instability of CB episodes in participants were in Scatter Plot, ABC, and The Observer XT procedure. This instability was higher for David, but was also evident for the other participants. Thus, when CB episodes are unstable, sporadic, and severe, as in the case of David, the information provided by the Scatterplot seems useful, using the ABC record only when events occurred. In this case, the Observer XT procedure did not seem effective. The ABC procedure was useful when continuously recording over time. Preferably, it should be conducted by an external observer, which involved a minimal effect on CB, although this methodology requires of human resources that are not always available to assisting agencies.

The Observer XT was useful for those participants who had frequent CB episodes throughout the day, as observed in Vicente, Ivo, and Jorge. Although it is difficult to implement because is time consuming, this method allowed us to get extensive information about events occurring in a natural setting.

ABC and The Observer XT records should be personalized. It was not assume that a CB (e.g., head banging) was affected by the environment in the same way for all individuals (Mudford et al., 2008), and probably for the same individual at two different instants. Therefore, before to carry out data collection through direct observation, we must operationally define CB and accurately specify antecedents and consequences provided by families or direct-caregiver staff during the interview (Dixon et al., 2012).

Regarding the timing assessment to provide a baseline, there is no criterion defining the number of sessions in which we must make a direct observation of behavior. Some authors (Franco, 1998) set a period of 2 or 3 weeks, and can be extended if necessary. In this study, the period was not sufficient in all cases, because the participants' behaviors considerably varied over time. As shown in ABC and The Observer XT comparative results, the baseline obtained with each of procedures differed. These differences can be explained by the instability of the episodes, rather than the procedure used.

Considering results of this study, theoretical implications and some advantages and disadvantages are recommended. Assessment is a milestone for planning behavioral intervention and reduce CB. For this reason, to choice an appropriated register instrument is required. Scatter Plot highlights being a practical, easy to record, in reporting the behavior stability. The ABC report the frequency and context in which behavior problems occur. In addition, one of the benefits of The Observer XT is the comprehensive analysis of the different topographies of each behavioral category. However, Scatterplot does not collect information on frequency, duration and behavioral context. ABC did not allow us re-check episodes because it is a live observation procedure. Therefore, relevant information in order to know the behavior context may unnoticed. Several studies recommend short experimental sessions to corroborate or refute the hypotheses derived from the ABC functional register (Hanley et al., 2003). Finally, The Observer XT was the most efficient procedure, but special training and cost-benefits analysis is required to implement it in clinical or educational settings.

This study has some methodological weaknesses to consider in future research. Only two out of four participants could be evaluated with the three behavioral observation procedures (Ivo and Jorge). This was because the lack of human resources and time commitment of direct caregiver staff. Furthermore, only in two cases an IOA was calculated using Observer XT because one participant (David) did not exhibit any CB during all observation periods.

In summary, taking into account the advantages and disadvantages posed by long-term behavioral observation registers and variability of behavior presented by people with severe ID, we propose the following guidelines to select the most appropriate procedure: (a) For infrequent behaviors that occur with severe intensity, we can use the Scatterplot and daily ABC record sheets only when an episode appears. (b) When behavior problems occur with high frequency and are unstable, daily record by the ABC method provides accurate information on the frequency. (c) When behavior problems occur with high frequency and are stable over time, The Observer XT is useful to provide comprehensive information on the different topographies of the behavioral categories. (d) Finally, if we are interested to know the duration of the behavior, the best choice seems The Observer XT, because it can achieve high accuracy of the detailed analysis of the duration of behavior. Further research with a larger number of participants and inter observers reliability would be appropriate in order to increase validity of this approach, focusing not only on behaviors' frequency and duration, but also on CB intensity.

## Author contribution

All authors participated on three tasks for this article: Data gathering, Data analysis, Writing and Reviewing.

### Conflict of interest statement

The authors declare that the research was conducted in the absence of any commercial or financial relationships that could be construed as a potential conflict of interest.
